# Clinical insights into circulating free-DNA in patients with bone sarcomas and ewing sarcoma

**DOI:** 10.1016/j.clinsp.2025.100661

**Published:** 2025-04-26

**Authors:** Veronica Aran, Amanda Santos Cavalcanti, Walter Meohas, Bruna Canteri, Jamila Alessandra Perini, Jesus Pino Minguez, João Antônio Matheus Guimarães, Vivaldo Moura Neto, Maria Eugênia Leite Duarte

**Affiliations:** a Laboratório de Biomedicina do Cérebro, Instituto Estadual do Cérebro Paulo Niemeyer, Rio de Janeiro, RJ, Brazil; b Divisão de Pesquisa, Instituto Nacional de Traumatologia e Ortopedia (INTO), Rio de Janeiro, RJ, Brazil; c Laboratório de Pesquisa de Ciências Farmacêuticas (LAPESF), Universidade do Estado do Rio de Janeiro (UERJ), Rio de Janeiro, RJ, Brazil; d Departamento de Ortopedia, Universidade de Santiago de Compostela, Santiago de Compostela, Galicia, Spain; e Instituto D'Or de Pesquisa e Ensino (IDOR), Rio de Janeiro, RJ, Brazil; f Laboratório de Morfogênese Celular (LMC), Instituto de Ciencias Biomédicas (ICB), Universidade Federal do Rio de Janeiro (UFRJ), Rio de Janeiro, Brazil

**Keywords:** Liquid biopsy, IDH2, TP53, Bone tumours

## Abstract

**Background and objectives:**

Sarcomas represent a heterogeneous group of malignancies characterized by varying clinical behaviors and treatment responses. Liquid biopsy has emerged as a promising non-invasive method for monitoring tumor dynamics by detecting actionable mutations in cancer patients. The emergence of circulating DNA as a non-invasive biomarker offers promising avenues for improving diagnostic accuracy and treatment monitoring in sarcoma patients.

**Methods:**

In this study, the authors employed mutation-specific droplet digital PCR (ddPCR) to analyze tumor-derived cell-free DNA, also known as circulating tumor DNA (ctDNA), belonging to plasma samples of sarcoma patients, aiming to characterize mutation profiles in the IDH2 and TP53 genes. Between July 2019 and June 2023, the authors collected and analyzed 38 samples from patients diagnosed with osteosarcoma, chondrosarcoma, or Ewing's sarcoma. Histopathological confirmation of diagnoses was performed, followed by ddPCR analysis on 36 valid plasma samples.

**Results:**

The results showed mutations in three out of thirty-six sarcoma patients. Patient 1 exhibited a 12.6 % mutant IDH2 (R172S) allele fraction, Patient 2 had a 0.27 % mutant TP53 (R175H), and Patient 3 showed a 17 % mutant IDH2 (R172K). Notably, Patients 1 and 2 were diagnosed with chondrosarcoma, while Patient 3 had osteosarcoma.

**Conclusions:**

The present study provided evidence for the feasibility of ctDNA detection in sarcoma patients, where mutations were found in IDH2 and TP53 genes, including a novel IDH2 mutation in osteosarcoma. The evaluation of ctDNA has the potential to transform clinical strategies in this challenging group of malignancies and this may be further confirmed in larger cohort studies. Continued research efforts are essential to optimize ctDNA detection methods and validate its utility across diverse sarcoma subtypes.

## Highlights

• Non-invasive ctDNA monitoring shows promise for sarcoma patient management.
• ddPCR identified IDH2 and TP53 mutations in bone sarcoma plasma samples.
• Liquid biopsy could improve bone sarcoma diagnosis.
• Study highlights the detection of a novel IDH2 mutation in osteosarcoma.

## Introduction

According to the most recent WHO classification of Soft Tissue and Bone Tumours, osteosarcoma is a malignant primary bone tumor characterized by the production of osteoid, and originating from primitive bone-forming mesenchymal cells.^1^ Primary chondrosarcomas encompass a heterogeneous group of malignant cartilage-forming tumors that arise in preexisting normal bone. Ewing Sarcoma is a highly aggressive malignancy categorized under undifferentiated small round cell sarcomas of bone and soft tissues, primarily occurring in children and young adults.^1^^,^^2^

Among osteogenic tumors, osteosarcoma is the most common bone malignancy, representing approximately 20 % of all primary bone tumors.^1^^,^^3^ It affects individuals under 20-years-old, with a second peak of incidence in adults over the age of 40.^1^^,^^4^ Osteosarcoma is more frequent in males and preferentially affects the knee joint, with the lungs being the main site of metastases.^1^^,^^2^^,^^4^ The 5-year survival rate is <30 % in patients with metastasis at initial diagnosis but exceeds 80 % with early diagnosis and appropriate treatment.^3^^,^^4^ Chondrosarcoma is the second most common primary bone tumor, accounting for approximately 20 %‒25 % of bone neoplasms. It arises from cartilage-producing cells and is more common in adult males.^1^^,^^5^ High-grade chondrosarcomas have a 10-year survival rate of <30 %.^5^ Conventional chondrosarcomas form a spectrum of diseases determined by the tumor's biological behavior ranging from relatively benign low-grade tumors or intermediate Atypical Cartilaginous Tumors (ACTs) to aggressive high-grade malignant tumors.^5^ Ewing's sarcoma is the third most frequent malignant bone tumor in children and young individuals accounting for approximately 16 % of bone neoplasms, but in young patients, it is the second most common tumor, presenting higher incidence in boys.^1^ With appropriate treatment, the 5-year survival rate can exceed 60 %, but it drops to <30 % in cases of recurrence or metastasis.^6^

The diversity and considerable overlap of characteristics among bone tumors can complicate accurate diagnosis and, consequently, appropriate personalized disease management. Therefore, detecting tumor-specific molecular alterations can facilitate precise diagnosis in challenging cases. Traditional cancer management approaches, such as tissue biopsies and surgery, are highly invasive procedures and have limitations, especially when repeated sampling is needed for disease monitoring.^7^ Traditional biopsies, performed directly on tumor tissue, remain essential for most cancer types and hold high diagnostic value.^8^ However, obtaining tissue samples can be challenging and pose risks to the patient. Tumor sampling is also compromised by tumor heterogeneity, as tumors can have different clonal cell populations in different intratumoral locations, also conventional biopsy might lead to tract contamination during the biopsy.^9^ In contrast, liquid biopsy is an innovative, minimally invasive procedure that allows the detection of tumor-related molecular changes through biological fluids such as blood, saliva, cerebrospinal fluid, pleural effusions and urine.^10^ Minimally invasive sampling can be performed at various stages of the disease, providing physicians with genetic information from both primary tumors and metastases. Furthermore, liquid biopsy can gather a more comprehensive representation of the entire tumor, including information from multiple sites, such as in the pituitary gland where the group identified, for the first time, mutant K-RAS both in the blood circulation (plasma) and matching tumor tissue.^11^

Circulating Tumor Cells (CTCs), circulating cell-free DNA (cfDNA), circulating tumor DNA (ctDNA) and Extracellular Vesicles (EVs) are sources of tumor biomarkers for liquid biopsy.^12^ The levels of cfDNA can vary significantly, with cancer patients often exhibiting levels that are 5 to 10 times higher than those of healthy individuals, and a portion of this fragmented DNA comes from the tumor itself and is referred to as ctDNA.^13^ There are various candidate genes implicated in the development of bone neoplasms that could be investigated by evaluating ctDNAs using liquid biopsy. Amary and colleagues described mutations in cartilaginous tumors, specifically somatic heterozygous isocitrate Dehydrogenase 1 (IDH1) hotspots (R132C and R132H) or IDH2 (R172S), which are not found in other mesenchymal tumors.^14^ Mutations in the gene Tumor Protein 53 (TP53) have been found in osteosarcoma, chondrosarcoma and Ewing sarcoma^15^^,^^16^ and mutations in IDH2 are rare events in osteosarcoma.^17^ Despite the evident benefits and applicability in other tumors such as melanoma, lung cancer,^18^ ovarian cancer,^19^ colorectal cancer^20^ among others, the application of liquid biopsy in musculoskeletal tumors is not yet well established. In the present study, the authors employed mutation-specific droplet digital PCR (ddPCR) to analyze circulating ctDNA from plasma samples of patients with osteosarcoma, chondrosarcoma and Ewing Sarcoma, with the goal of characterizing mutation profiles in the IDH2 and TP53 genes. By undertaking this approach, the authors aim to investigate the feasibility of detecting ctDNA mutations in the plasma belonging to bone sarcomas and Ewing sarcoma, addressing a critical gap in research. This effort has the potential to contribute to future diagnostic and monitoring strategies, ultimately enhancing patient care and outcomes in this challenging field.

## Materials and methods

### Study type and population

This is a cross-sectional study conducted with samples obtained from July 2019 to June 2023 at the National Institute of Traumatology and Orthopaedics in Rio de Janeiro, Brazil. All patients with a clinical suspicion of osteosarcoma, chondrosarcoma, or Ewing sarcoma were screened for eligibility. Histopathological confirmation of one of these three types of tumors occurred before cfDNA extraction. Additionally, participants were required to have an indication for a surgical approach (tumor biopsy or main surgery) of the primary tumor or a recurrent lesion. Patients undergoing chemotherapy treatment or with other active malignant diseases were excluded. In addition, patients with cfDNA levels below the limit of detection (cfDNA < 0.25 ng/uL) were also excluded from the ddPCR analysis.

Ethical approval was obtained from the National Institute of Traumatology and Orthopaedics’ Human Ethics Committee (ref:3.502.865), and all participants or their legal representatives provided written informed consent. The STROBE checklist was used to elaborate this article.

### Sample collection and processing

Peripheral blood samples (8 to 15 mL) were collected through venous access in Ethylenediaminetetraacetic Acid (EDTA) tubes before the administration of anesthesia for surgery and stored at 4 °C until processing, which occurred a maximum of two hours after collection. Processing was carried out in two steps to ensure a cell-free plasma. First, the whole blood was centrifuged at 1200 × *g* for 10 min to remove cells. The plasma was then transferred to a microtube and centrifuged at 16,000 × *g* for 15 min at 4 °C to eliminate any remaining cells. The plasma was stored at −80 °C until the DNA extraction step. The use of plasma (rather than serum) in addition to the two centrifugation processes described here are important steps to avoid contaminating the sample with genomic DNA released by blood cells, thus enhancing the sensitivity of cfDNA analysis.^21^^,^^22^

### Cell-free DNA extraction and ddPCR analysis

Cell-free DNA extraction was carried out using 1 mL of plasma in the Maxwell® RSC automated instrument (Promega Corporation, USA), following the manufacturer's instructions. The cfDNA samples were then accurately quantified using the Quantus™ Fluorometer (Promega).

Molecular analysis was performed to investigate IDH2 and TP53 mutations using ddPCR from Bio-Rad, following the previously described protocol for cell-free DNA extraction.^23^ ddPCR reactions were conducted in a 20 μL volume containing 1 × ddPCR Supermix (no dUTP; Bio-Rad, CA, USA), 900 nmoL/L primers, 250 nmoL/L probes, and up to 8 μL of DNA with RNAse-free water. Specifically, 5 ng of cfDNA from plasma were tested in each reaction. The following ddPCR multiplex mutation assays (i.e., assays that detect both wild-type and mutant sequences) were employed: IDH2 p.R172S (dHsaMDS2515400, Bio-Rad), IDH2 p.R172K (dHsaMDV2010059, Bio-Rad), and TP53 p.R175H (dHsaMDV2010105, Bio-Rad) using the QX200 Droplet Digital PCR System (Bio-Rad). Amplifications were performed with the cycling conditions: 1 cycle at 95 °C for 10 min, 40 cycles at 94 °C for 30 s, 55 °C for 1 min, followed by 1 cycle at 98 °C for 10 min. Absolute quantification of mutant and wild-type alleles was performed using QuantaSoft Analysis Software (Bio-Rad). Allele detection thresholds were established based on the signal from empty droplets (negative control). The system's software allows for the clear delineation of thresholds between positive and negative droplet clusters, according to the manufacturer's guidelines (Bio-Rad). To ensure accuracy, the authors used non-template controls and cfDNA extracted from plasma samples of healthy individuals. This was done to verify that the ddPCR mutation assays specifically detected cancerous mutations and not mutations of non-cancerous origin.

## Results

### Patient characteristics

**Fig. 1:**
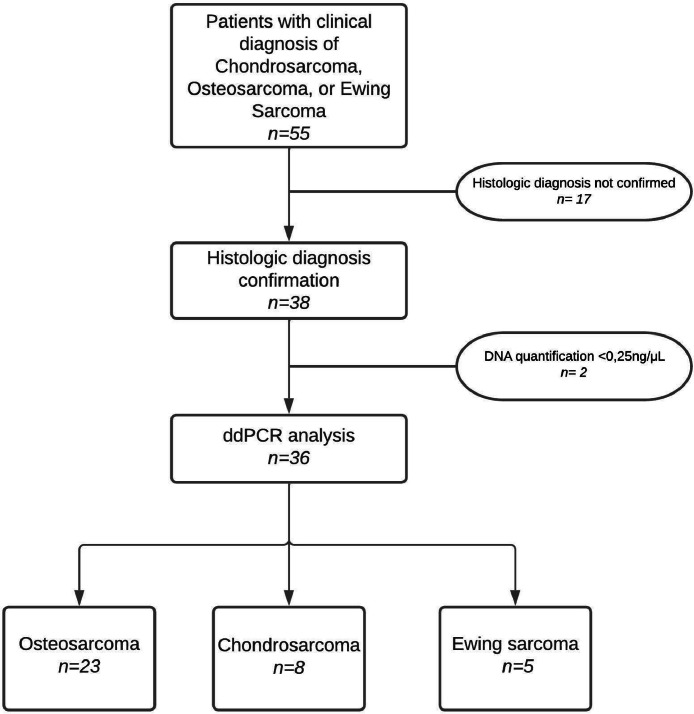
Flow chart summarizing study participants.

Over the course of one and a half years, 55 samples were collected from patients clinically diagnosed with osteosarcoma, chondrosarcoma, or Ewing's sarcoma. After histopathological confirmation of the diagnoses, cfDNA was extracted from 38 individuals in order to proceed with the ddPCR analysis. Two patients showed insufficient amounts of cfDNA (cfDNA < 0.25 ng/uL), therefore they were excluded from the analysis. Fig. 1 summarizes the study's flowchart.

Patient demographics and clinicopathological characteristics are summarized in Table 1. The follow-up period was defined as the time between diagnosis and either the date of death or the last outpatient visits to the hospital.

**Table 1:** Baseline characteristics of participants included in the ddPCR analyses (*n* = 36).

	Osteosarcoma (*n* = 23)	Chondrosarcoma (*n* = 8)	Ewing sarcoma (*n* = 5)
**Median age (range)**	15 (6 – 61)	45 (40 – 70)	9 (8 – 19)
**Gender (F/M)**	10/13	3/5	3/2
**Metastatic disease (n)**	12	3	1
**Site of metastasis**			
Lungs	12	1	1
Skip metastasis	0	1	0
Lungs and skull	0	1	0
**Follow-up in months mean ± SD (range)**	23.1 ± 19.7 (1.1 – 86.9)	28.8 ± 21.1 (6.2 – 66.3)	8.7 ± 5.0 (2.4 – 15.0)
**Death (n)**	6	2	2
**Months from diagnosis to death mean ± SD (range)**	20.6 ± 16.6 (5.2 – 50.1)	26.8 ± 21.4 (11.6 – 41.9)	13.7 ± 1.8 (12.5 – 15.0)

F, Female; M, Male.

### Analyses of cfDNA from plasma samples

**Fig. 2:**
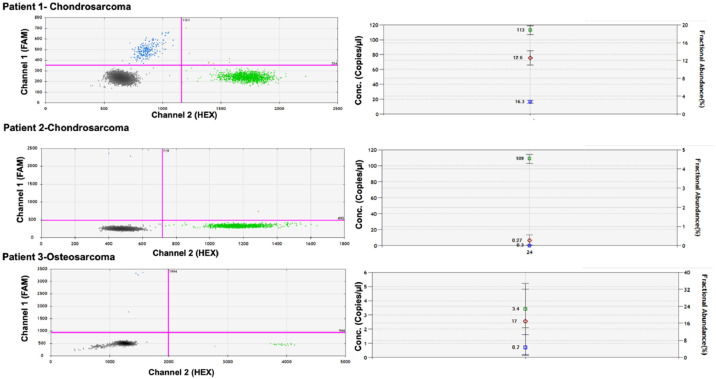
Two-dimensional scatter plot illustrating the four clusters obtained ctDNA analysis from plasma belonging to three patients. Blue dots refer to mutant samples and green dots represent wild-type alleles. Each patient shows a graph regarding mutation analysis and graph regarding mutant allele frequency (number of mutant droplets over total count of mutant plus wild-type droplets as described in the methods’ section). In Patient 1, the percentage of mutant IDH2 (R172S) relative to wild-type sequences in plasma was 12.6 %, whereas Patient 2 exhibited 0.27 % mutant TP53 (R175H), and Patient 3 showed 17 % mutant IDH2 (R172K). In the graph, the fluorescence of channel 1 (FAM) is plotted against the fluorescence of channel 2 (HEX) for each droplet, distinguished by color (blue, black, orange, or green). The drops are categorized into the following groups: FAM negative, HEX negative (doubly negative drops, marked in black); FAM positive, HEX negative (positive drops indicating mutation, marked in blue); FAM negative, HEX positive (positive drops indicating wild-type sample, marked in green); FAM positive, HEX positive (doubly positive drops containing both wild-type and mutated DNA, labeled in orange). The graphs and the absolute quantification of mutant and wild-type alleles were obtained using the QuantaSoft Analysis Software (Bio-Rad).

Analysis of cfDNA from plasma samples involved pre-surgery collection followed by mutation-specific ddPCR analysis using Bio-Rad assays, as described in the methods section. These assays identify both wild-type and mutant forms of the gene in the patient's plasma, employing liquid biopsy techniques. Thirty-six patients underwent ddPCR analysis, revealing mutations in three individuals. Two-dimensional scatter plots (Fig. 2) illustrate that in Patient 1, the percentage of mutant IDH2 (R172S) relative to wild-type sequences in plasma was 12.6 %. Patient 2 exhibited 0.27 % mutant TP53 (R175H), while Patient 3 showed 17 % mutant IDH2 (R172K). Patients 1 and 2 were diagnosed with chondrosarcoma, whereas Patient 3 had osteosarcoma. The patients with Ewing Sarcoma were wild-type for the genes tested. Fig. 2 displays distinct mutation frequencies among the three patients, reflecting varying allele frequencies.

### Clinical, radiographic, and histologic features of patients with liquid biopsy mutations

**Fig. 3:**
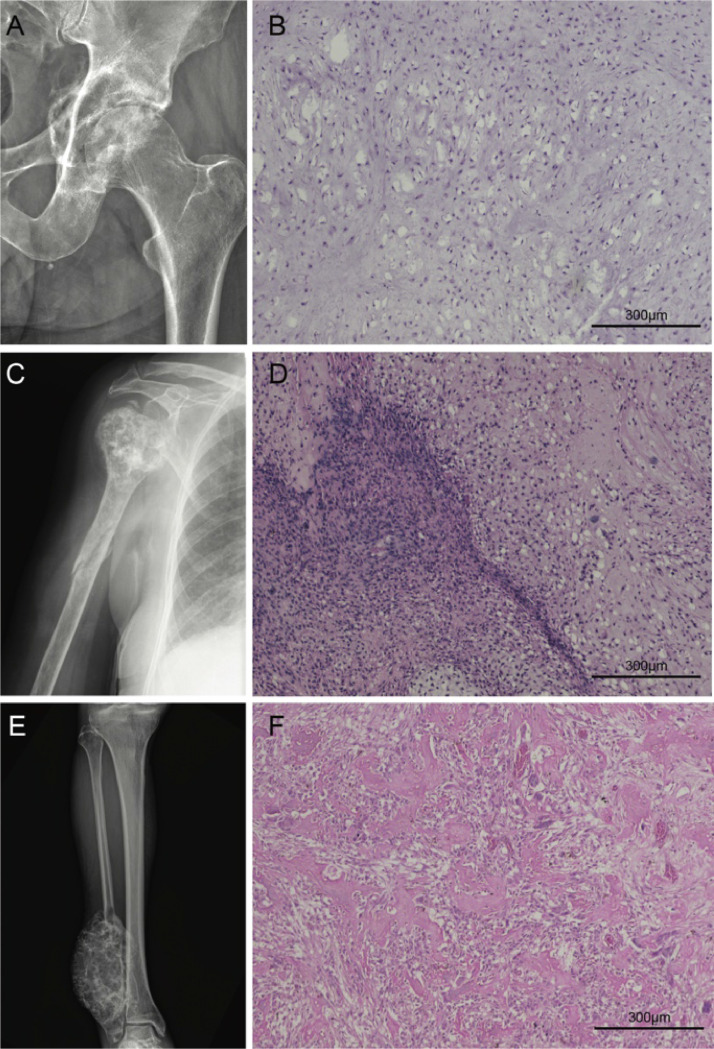
Radiographic and histologic features of patients with gene mutations. (A) Large permeative lytic lesion located in the proximal third of the humerus with amorphous calcification within the tumor matrix, irregular cortical destruction, and soft tissue extension. A pathological fracture is evident in the diaphysis. (B) Histopathological findings confirmed the diagnosis of high-grade chondrosarcoma with areas showing dedifferentiation to high-grade undifferentiated pleomorphic sarcoma. The chondrosarcoma features (top right) include pleomorphic chondrocytes embedded within a chondroid eosinophilic matrix. In contrast, the regions representing undifferentiated pleomorphic sarcoma (left) exhibit high cellular density. Unlike the chondrosarcoma component, there is a notable absence of a cartilage-like matrix, with cells embedded in a dense stroma. (C) Lytic lesion with poorly defined margins within the acetabulum, displaying irregular erosion of the cortical bone and internal calcifications within the tumor matrix. (D) Histological findings confirmed the diagnosis of grade II conventional chondrosarcoma characterized by moderate cellularity and nuclear atypia. Atypical chondrocytes are observed in lacunae surrounded by a bluish-gray cartilage matrix. (E) Large infiltrating lesion distorting the normal bone structure in the distal fibula with lytic areas of extensive bone destruction and areas of bone formation, typically present in osteosarcoma. (F) Histopathological findings confirmed the diagnosis of classic central osteosarcoma displaying malignant osteoblasts producing irregularly deposited eosinophilic, unmineralized bone matrix (osteoid).

Patient 1 was a 70-year-old woman who presented with a chondrosarcoma relapse at the right proximal humerus (Fig. 3A). She had a three-month history of pain and underwent a biopsy, which confirmed a diagnosis of high-grade chondrosarcoma (Fig. 3B). Subsequently, she underwent resection surgery with placement of an endoprosthesis. Fifteen months later, she experienced a recurrence and underwent another resection surgery. Within 29 months, she had a second local recurrence, and it was during this period that the blood sample was collected. By this time, the patient had developed lung metastases and died five months later.

Patient 2, a 62-year-old man, was diagnosed with chondrosarcoma of the left acetabulum (Fig. 3C). He had reported pain for seven months before his clinic visit. A biopsy within one month confirmed grade II dedifferentiated central chondrosarcoma (Fig. 3D). Three months later, he underwent a hemipelvectomy. One year post-surgery, the patient experienced a local recurrence of the tumor, which was diagnosed as grade III dedifferentiated chondrosarcoma. The dedifferentiated component of the lesion included high-grade undifferentiated pleomorphic sarcoma and osteosarcoma, comprising approximately 10 % of the tumor volume. He died shortly after the diagnosis due to complications related to the neoplasia.

Patient 3, a 43-year-old man, initially presented with grade III central osteosarcoma in the right fibula (Fig. 3E and F). He received neoadjuvant chemotherapy followed by transtibial amputation of the right lower limb. Eleven months post-surgery (December 2023), he was diagnosed with lung metastasis. At the time of final data collection for this study, four months later, the patient was still alive.

## Discussion

In the present study, ddPCR was utilized to detect mutations in the IDH2 and TP53 genes in plasma samples from patients with three types of bone sarcomas: an aggressively malignant bone-forming tumor (osteosarcoma), a malignant bone tumor-producing cartilaginous matrix (chondrosarcoma), and a distinctive small round cell sarcoma of bone (Ewing sarcoma). The analysis included 38 plasma samples from patients with these three types of bone sarcomas. The authors identified mutations in three out of 36 valid samples (8.3 %), with two patients (Patient 1 with chondrosarcoma and Patient 3 with osteosarcoma) having mutations in IDH2, and one patient with grade II conventional chondrosarcoma (Patient 2) having a mutation in TP53. Patient 1, despite multiple surgeries for chondrosarcoma, exhibited a progressive disease course with recurrent local tumors and lung metastases, correlating with the presence of the IDH2 mutation. The fact that chondrosarcoma may metastasize to the lungs has also been described.^24^ Patient 2 also experienced disease recurrence and succumbed to chondrosarcoma within a year post-surgery. In contrast, Patient 3 showed evidence of disease progression but remained alive at the study's conclusion. IDH1/2 ctDNA has previously been correlated with relapse risk,^25^ and the TP53 p.R175H mutation has been associated with poor survival.^25^

Research on detecting IDH2 mutations in plasma from patients with chondrosarcoma, osteosarcoma, and Ewing Sarcoma is currently limited. For example, a study published in 2021 explored the potential of ctDNA as a biomarker for central chondrosarcoma. The researchers analyzed plasma samples from patients and identified mutations in the IDH2 gene, with a frequency of approximately 10 %,^26^ just slightly lower than what the authors found (*n* = 1/8; 12.5 % of chondrosarcoma cases). When the authors compare the present data to analysis performed in tumor samples an average of 12.1 % of IDH2 mutations have been described in chondrosarcoma tumor cases, being higher among grade III when compared to Grade II.^27^^,^^28^ Interestingly, while some studies have investigated IDH1/2 mutations in osteosarcoma, only one study reported the novel detection of the IDH2 R172S mutation in three out of 12 osteosarcoma patients (25 %) using direct DNA sequencing.^17^ In the present study, the authors detected for the first time the mutation IDH2 R172K, which opens a window of questions regarding the events that connect IDH2 mutations and osteosarcoma development.

The detection of specific TP53 mutations, such as R175H, in the plasma of chondrosarcoma patients has not been extensively documented in the scientific literature. The authors detected 12.5 % of TP53 mutations in the chondrosarcoma plasma samples tested (*n* = 1/8). The mutation levels described are approximately 20 % when tumors are analyzed (not plasma).^29^ Tumor samples exhibit a significantly higher number of mutation frequency compared to plasma samples, because tumor tissue represents a concentrated collection of mutated cells, whereas plasma contains only a small fraction of ctDNA shed by tumor cells. As a result, plasma analysis has a lower overall mutation detection rate. The identification of specific ctDNA mutations provides valuable insights into tumor detection and dynamics since different bone tumor-associated mutations could be screened in a multiplex manner improving the probability of detecting ctDNAs according to each tumor type.^30^ Recently, a meta-analysis study utilized individual patient data to analyze the clinical and prognostic association of IDH1/2 mutations in chondrosarcoma patients compared to those without mutations.^27^ The authors concluded that IDH1/2 mutations could serve as distinct prognostic biomarkers, improving the accuracy of outcome predictions and aiding in the creation of personalized treatment plans. These mutations exhibit unique features in chondrosarcomas compared to non-mutated cases, making them valuable independent prognostic indicators, which could guide the development of suitable treatment strategies.^27^ Additionally, since tumor-derived DNA levels have been linked to tumor burden and disease progression across various cancer types, a similar relationship may be relevant for bone tumors.^31^^,^^32^ The observed differences in allele frequencies among patients in the present study could reflect underlying heterogeneity in tumor biology related to bone tumor burden.

Molecular biomarkers play a crucial role in enhancing cancer diagnosis, prognosis, and treatment by facilitating early detection, monitoring recurrences, and guiding treatment strategies.^25^ The implications of IDH mutations for cancer development and therapy have been previously discussed,^33^ however there is still much work to be done regarding investigating the mutations described here and their connection to bone sarcoma clinical management. Advances in sequencing technologies, such as Next-Generation Sequencing (NGS), have significantly improved diagnostic accuracy and clinical decision-making by providing comprehensive whole-genome analyses, thus advancing personalized medicine.^25^ Furthermore, highly sensitive techniques like ddPCR, used in this study, combined with assays capable of detecting alterations at variant allele frequencies below 0.5 %, have markedly improved ctDNA detection. This is particularly important given ctDNA's low concentration in plasma, as it allows for the early identification of tumor events and reduces the likelihood of false negatives.^25^ However, the clinical significance of low allele frequency mutations, such as the 0.27 % observed in Patient 2, is a field under constant debate.^31^ Consequently, the findings from the present study underscore the need for further investigation to establish thresholds for actionable mutations and to assess the impact of low variant allele frequencies in bone sarcomas.

The identification of specific mutations in circulating DNA provides insights into the early detection of tumors and their dynamics, having potential prognostic implications, especially after surgery, since ctDNA detection already indicates the chance of recurrence in certain tumors such as in breast, colon, and lung tumors.^34^, ^35^, ^36^, ^37^ Monitoring ctDNA mutations using multiple sample collections may offer early detection of recurrence, particularly in cases where conventional imaging or clinical assessments may be inconclusive. In fact, levels of mutant ctDNA were shown to vary according to cancer type, stage, tumor burden, sites of tumor shedding, and treatment status.^38^ It has also been reported that IDH mutations detected in the primary chondrosarcoma tumor persist in local recurrences and metastases derived from that tumor, indicating its potential role as an initiating event in the disease.^39^ Although primary tumor biopsy will continue to be the definitive method for diagnosing sarcoma, cfDNA testing presents significant benefits that make it worth investigating further as an additional tool for diagnostics, prognosis, and disease monitoring.^40^

This study's limitations include the relatively small sample size, although in bone sarcoma studies the sample sizes are usually small. In addition, the heterogeneity of sarcoma subtypes may affect the generalizability of findings. Unfortunately, the exclusion of 2 samples due to insufficient DNA quantity for digital PCR analysis, and the exclusion of 17 samples that did not have histologic diagnosis confirmation, also affected the sample size. Larger multicentre studies are essential to confirm these results and address these limitations. Despite promising results, challenges such as the need for standardization across different sarcoma subtypes remain. Future research should focus on expanding the cohort size to validate findings across broader patient populations and refine the clinical utility of cfDNA analysis in sarcoma management. Integration with other biomarkers and genomic profiling with ctDNA analysis techniques could enhance the precision of mutation detection, improve patient outcomes, and explore its utility in guiding therapeutic decisions in sarcoma management.

## Conclusion

In conclusion, the present study demonstrates the utility of mutation-specific ddPCR for analyzing circulating DNA in patients with sarcomas. By providing early insights into tumor dynamics, ctDNA analysis holds promise as a non-invasive tool for personalized medicine strategies in this challenging group of malignancies. The detection of low mutant allele frequencies, even below 0.5 %, underscores the potential of ctDNA as a biomarker for early disease detection. Furthermore, the authors present, for the first time, the detection of the IDH2 R172K mutation in the plasma of an osteosarcoma patient, a result that should be further studied in a larger patient cohort. Continued research efforts are essential to optimize assay methodologies and establish standardized protocols for routine clinical use, thus guiding future research directions and advancing the field of liquid biopsy in oncology.

## Institutional review board statement

Ethical approval was obtained from the National Institute of Traumatology and Orthopedics (INTO, Rio de Janeiro, Brazil) Human Ethics Committee (ref: 3.502.865).

## Informed consent statement

All participants or their legal representatives provided written informed consent.

## Funding

This study was funded by the Brazilian Agency “Fundação de Amparo à Pesquisa do Rio de Janeiro” (FAPERJ 25191).JAP was supported by the Brazilian agencies FAPERJ E-26/210.949/2021 (which funded the publication fees of this work), 10.13039/501100003593Conselho Nacional de Desenvolvimento Científico e Tecnologico (CNPq, grant number 309065/2021-6) and UERJ (Prociencia 2023-2026).

## Declaration of competing interest

The authors declare that they have no known competing financial interests or personal relationships that could have appeared to influence the work reported in this paper.

## Data Availability

Full data will be provided by the corresponding author upon reasonable request.
